# Administration of chromium picolinate and meloxicam alleviates regrouping stress in dairy heifers

**DOI:** 10.5713/ab.24.0104

**Published:** 2024-04-26

**Authors:** Da Jin Sol Jung, Jaesung Lee, Do Hyun Kim, Seok-Hyeon Beak, Soo Jong Hong, In Hyuk Jeong, Seon Pil Yoo, Jin Oh Lee, In Gu Cho, Dilla Mareistia Fassah, Hyun Jin Kim, Mohammad Malekkhahi, Myunggi Baik

**Affiliations:** 1 Department of Agricultural Biotechnology and Research Institute of Agriculture and Life Science, College of Agriculture and Life Sciences, Seoul National University, Seoul 08826, Korea; 2 Institutes of Green Bio Science Technology, Seoul National University, Pyeongchang 25354, Korea

**Keywords:** Chromium, Displacement Behavior, Heifer, Meloxicam, Regrouping Stress

## Abstract

**Objective:**

This research investigated the effect of administering chromium (Cr) and meloxicam (MEL) on growth performance, cortisol and blood metabolite, and behaviors in young, regrouped heifers.

**Methods:**

Fifty Holstein dairy heifers (body weight [BW] 198±32.7 kg and 6.5±0.82 months of age) were randomly assigned to non-regrouped group or four regrouped groups. Non-regrouped animals were held in the same pen throughout the entire experimental period (NL: non-regrouping and administration of lactose monohydrate [LM; placebo]). For regrouping groups, two or three heifers maintained in four different pens for 2 weeks were regrouped into a new pen and assigned to one of four groups: regrouping and LM administration (RL); regrouping and Cr administration (RC); regrouping and MEL administration (RM), and regrouping and Cr and MEL administration (RCM). LM (1 mg/kg BW), Cr (0.5 mg Cr picolinate/kg dry matter intake), and MEL (1 mg/kg BW) were orally administered immediately before regrouping. Blood was collected before regrouping (0 h) and at 3, 9, and 24 h and 7 and 14 d thereafter. Behaviors were recorded for 7 consecutive days after regrouping.

**Results:**

Average daily gain was lower (p<0.05) in RL than NL heifers, but was higher (p<0.05) in RM, RC, and RCM than RL heifers. RL heifers had higher (p<0.05) cortisol than NL heifers on d 1 after regrouping. The cortisol concentrations in RC, RM, and RCM groups were lower (p<0.05) than in RL treatment 1 d after regrouping. Displacement behavior was greater (p<0.05) in RL group than all other groups at 2, 3, and 6 d after regrouping.

**Conclusion:**

Regrouping caused temporal stress, reduced growth performance, and increased displacement behavior in heifers. Administering Cr and MEL recovered the retarded growth rate and reduced displacement behavior, thereby alleviating regrouping stress.

## INTRODUCTION

Introducing one individual animal, or an entire group of animals, into an established herd or making a new herd affects social relationships [1,2]. Regrouping of cattle according to age, live weight, or production stage, and after weaning, is a common practice to increase the efficiency of the feeding process and group homogeneity [3–5]. Regrouping of cows changes the hierarchical order among them, forcing the cows to reestablish social relationships through physical and nonphysical interactions and exacerbating aggressive and submissive behaviors [6,7]. Regrouped heifers suffer from a high level of stress due to competition for feed and lying places [8]. Regrouping stress alters the physiology of dairy cattle: newly introduced cattle have higher cortisol concentrations than non-regrouped cattle [9,10]. Therefore, regrouping can adversely affect animal welfare, productivity, and health, and farm profitability [11].

Nonsteroidal anti-inflammatory drugs, including meloxicam (MEL), have been used to alleviate stress caused by dehorning, castration, and transport. For example, MEL reduced blood cortisol concentrations after dehorning [12] and castration of calves [13]. Chromium (Cr) supplementation may be another viable approach to reduce stress by increasing insulin sensitivity [14]. For instance, Cr supplementation reduced circulating cortisol levels and increases average daily gain (ADG) and feed intake in transported calves [14], and increased ADG in heat-stressed dairy calves [15]. However, the effects of Cr and MEL on regrouping stress in cattle have not been investigated. This study was performed to evaluate the effects Cr, MEL, and their combination on growth performance, blood cortisol and metabolite profiles, and behavioral responses in regrouped Holstein heifers.

## MATERIALS AND METHODS

The present study was carried out at a heifer-specific farm located at 825-7 in Mussuri, Dangjin, South Korea. All experimental procedures involving animals were approved by the Seoul National University Institutional Animal Care and Use Committee (SNUIACUC: SNU-180717-2) and conducted following the Animal Experimental Guidelines of the SNUIACUC.

### Animals, regrouping, and feeding

**Figure 1:**
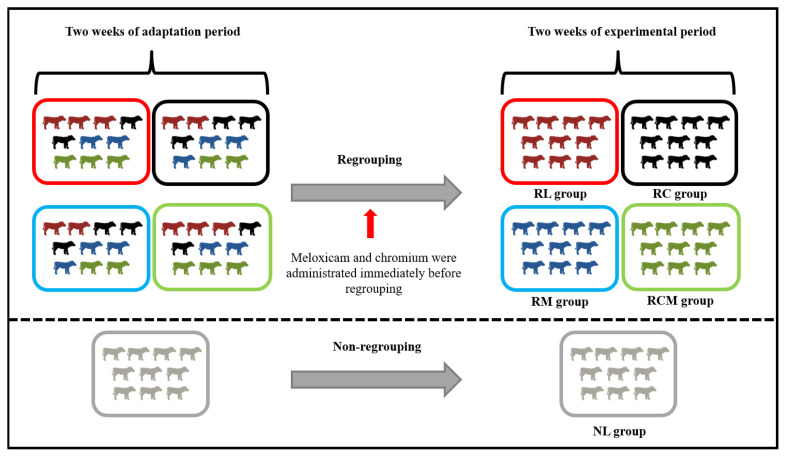
Regrouping strategy for the Holstein heifers. Fifty Holstein dairy heifers were randomly assigned to one non-regrouped group and four regrouped groups. The non-regrouped animals (n = 10) were held in the same pen during the entire experimental period (NL: no regrouping and administration of lactose monohydrate). Two or three heifers held in four different pens for 2 weeks were regrouped into a new pen and assigned to one of four groups (n = 10/group): regrouping and administration of lactose monohydrate (RL); regrouping and administration of Cr (RC); regrouping and administration of meloxicam (MEL) (RM); and regrouping and administration of both Cr and MEL (RCM).

Fifty Holstein dairy heifers (body weight [BW]: 198±32.7 kg; 6.5±0.82 months of age) were randomly assigned to a non-regrouped group and four regrouped groups. Two or three heifers from each regrouped group were placed in four different pens (10.0×15.0 m^2^; 10 animals per pen) with a feed alley (1.0×0.5 m^2^) and kept for 2 weeks (Figure 1). The non-regrouped animals (n = 10) were kept in the same pen during the entire experimental period (NL: non-regrouping and administration of lactose monohydrate [LM; placebo]). The two or three heifers from the regrouped groups grown in the four different pens were regrouped into a new pen, as shown in Figure 1 and assigned to four groups (n = 10/group): regrouping and administration of lactose monohydrate (RL); regrouping and administration of Cr (RC); regrouping and administration of MEL (RM); and regrouping and administration of Cr and MEL (RCM). Investigators were not blind to treatments. Oral doses of D-lactose monohydrate, Cr, and MEL were prepared as suspensions in 15 mL of water in a 20-mL dosing syringe: D-lactose monohydrate was prepared with 1 mg/1 kg BW of D-lactose monohydrate (Avantor Performance Materials); the Cr was prepared with 0.5 mg Cr picolinate/kg dry matter intake (Samjo; Cr content = 10.47%); the MEL was prepared at a dose of 1 mg/kg BW (MOBIC CAP, 15 mg MEL/270 mg of capsule content; Boehringer Ingelheim, Ingelheim, Germany). An oral dose was administered immediately before regrouping.

All heifers were fed a total mixed ration (TMR) during the experimental period. The TMR was formulated according to the NRC [16] recommendations for 6 to 7-month-old dairy heifers (Supplementary Tables S1, S2). The dry matter, crude protein, ether extract, crude fiber, and ash contents of the TMR were determined using analytical methods provided by the Association of Official Analytical Chemists (AOAC) [17]. The neutral detergent fiber and acid detergent fiber contents of the TMR were analyzed using a sequential method in an ANKOM200 fiber analyzer (Ankom Technology, Macedon, NY, USA), as described in Van Soest et al [18]. The fixed amount of TMR was offered at 08:00 and 14:00 h daily to each pen. Group intake was recorded daily by weighing the offered feed; no feed was refused in any group. The heifers were weighed and measured on the same 2 consecutive days each week at 07:00 h, and their weight was used to calculate the ADG. The heifers had free access to freshwater. During the experimental period, the average temperature and humidity were 21.5°C and 78.4%, respectively, and no precipitation was observed.

### Blood collection and analysis

Blood was collected externally from the jugular vein using a syringe at −1 h, 3 h, 9 h, 1 d, 7 d, and 14 d after regrouping; 10 mL of blood was collected into ethylenediaminetetraacetic acid (EDTA) vacutainers (K2E; BD Biosciences, Franklin Lakes, NJ, USA) to obtain plasma, and 10 mL was collected into non-heparinized vacutainers (SST II Advance; BD Biosciences, USA) to obtain serum. The EDTA tubes were stored on ice and centrifuged for 15 min at 1,800×g (4°C). The non-heparinized vacutainers were stored at room temperature for 30 min and centrifuged for 15 min at 1,800×g (4°C). The plasma and serum were stored at −70°C for the enzyme-linked immunosorbent assay (ELISA) and metabolite analyses. Plasma cortisol was analyzed using a cortisol salivary HS ELISA kit (SLV4635; DRG). Serum glucose and non-esterified fatty acids (NEFAs) were analyzed using commercial kits and an automated analyzer (Cobas 8000 C702 auto analyzer; Roche Diagnostics GmbH, Indianapolis, IN, USA). The Roche GLUC2 kit was used to analyze serum glucose. The Wako NEFA-HR2 kit (Wako Chemicals, Richmond, VA, USA) was applied for the serum NEFA analysis. The analytical methods used were validated in previous reports from our laboratory [19,20].

### Behavioral recording

Heifer behavior was recorded continuously for 7 consecutive days (d 0 to 6 after grouping) using video cameras (EZVIZ C3S, Los Angeles, CA, USA). The videos were stored on a 128 GB micro SD memory card (Sony Corp., Tokyo, Japan). The durations of lying and eating and the frequencies of eating, lying, and displacement were measured by viewing fast-forward video. Detailed descriptions of the behaviors are provided in Supplementary Table S3.

### Statistical analysis

A power analysis was performed to calculate the sample size for primary outcome variables in heifers. Based on data from Chibisa et al [21], with 85% confidence and 80% power, 10 animals per treatment group were needed to detect differences. All data were assessed for normality using the UNIVARIATE procedure in SAS (SAS Institute). Data that were not normally distributed were transformed logarithmically. A completely randomized design was used and the data were analyzed by the repeated-measures MIXED procedure in SAS. The experimental unit was individual heifer. The statistical model included the fixed effects of treatment, sampling time, and treatment×time interaction, and the random effect of animal. Sampling time was treated as a repeated measure. Three variance-covariance structures (auto-regressive type 1, compound symmetry, and Toeplitz) were tested, and the covariance structure that minimized Schwarz’s Bayesian information criterion was chosen. Initial BW was used as a covariate in the analysis of final weight. Growth performance data were analyzed using the above model without the time effect. Tukey-Kramer test was applied to account for multiple comparisons. A p-value ≤0.05 was considered significant, and 0.05<p≤0.10 indicated a trend.

## RESULTS

### Growth performance, blood cortisol and metabolites

The ADG was lower (p<0.05) in RL heifers than in NL heifers, and it was greater (p<0.05) in the RC, RM, and RCM heifers than in RL heifers (Table 1).

**Figure 2:**
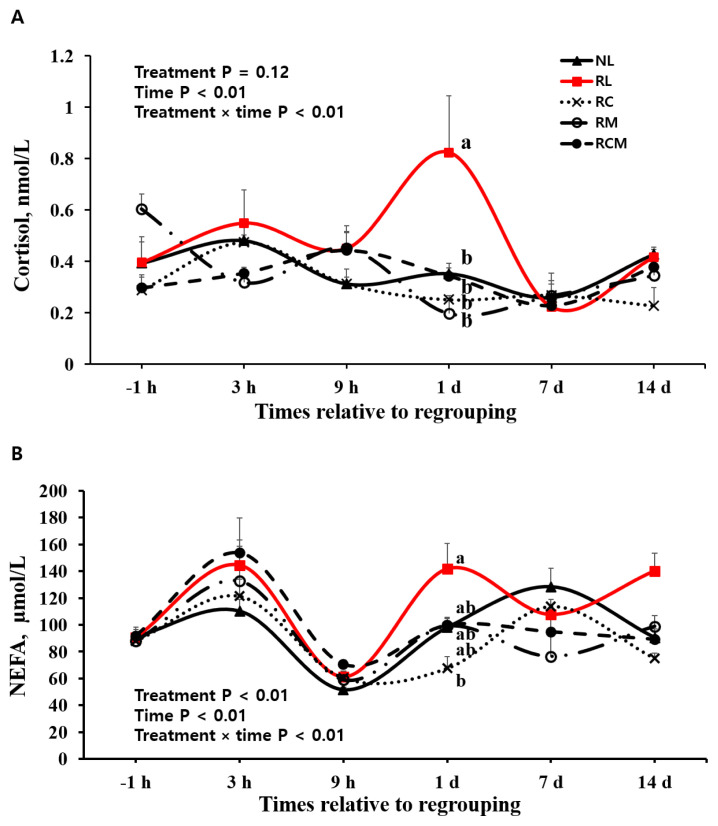
Effect of regrouping and administration of chromium and meloxicam on circulating cortisol and non-esterified fatty acids (NEFA) concentrations in Holstein heifers. NL, no regrouping and administration of lactose monohydrate; RL, regrouping and administration of lactose monohydrate; RC, regrouping and administration of chromium; RM, regrouping and administration of meloxicam; and RCM, regrouping and administration of both chromium and meloxicam. Values are means+standard error of mean (n = 10/group). ^a,b^ Means with different superscripts at each time point differ at p<0.05 (Tukey-Kramer test).

A treatment×time effect was detected (p<0.01) for plasma cortisol concentration (Table 2; Figure 2a). The RL heifers had a higher (p<0.05) cortisol concentration (0.82 nmol/L) than NL heifers (0.40 nmol/L) 1 d after regrouping, whereas cortisol concentrations were not different among the treatments at the other time points. Cortisol concentrations were lower (p<0.05) in the RC, RM, and RCM treatments than in the RL treatment 1 d after regrouping.

Circulating glucose concentrations differed (p<0.01) among treatments: they were higher in RCM heifers than in RM and RC heifers (Table 2). But, these did not differ between NL and RL heifers.

A treatment effect was detected (p<0.01) for the serum NEFA concentrations (Table 2). The RL group had higher (p<0.05) NEFA concentrations than the NL, RC, and RM groups. A treatment×time effect was observed (p<0.01) for serum NEFA concentrations (Table 2, Figure 2b). The RC group had a lower (p<0.05) NEFA concentration than the RL group 1 d after regrouping, although this difference was not detected at the other time points (Figure 2b).

### Behavioral observations

The duration and frequency of eating and lying were not different (p≥0.12) among the treatment groups (Table 3).

**Figure 3:**
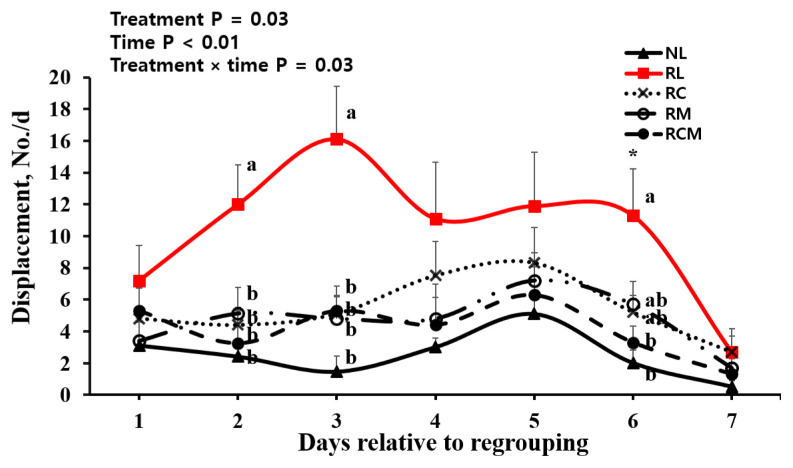
Effect of regrouping and administration of chromium and meloxicam on displacement behavior in Holstein heifers. NL, no regrouping and administration of lactose monohydrate; RL, regrouping and administration of lactose monohydrate; RC, regrouping and administration of chromium; RM, regrouping and administration of meloxicam; and RCM, regrouping and administration of both chromium and meloxicam. Values are means+standard error of mean (n = 10/group). ^a,b^ Means with different superscripts at each time point differ at p<0.05 (Tukey-Kramer test).

Treatment effect was observed (p = 0.03) for displacement behavior (Table 3). The number of displacements was 3.5-fold higher (p<0.05) in RL heifers (9.42/d) than in NL heifers (2.64/d), but the difference was not significant (p>0.05) among the NL, RC, RM, and RCM heifers. A treatment×time effect was observed (p = 0.03) for displacement behavior (Figure 3). Displacement behavior was greater in the RL group than in all other groups at 2, 3, and 6 d, but not at 1, 4, 5, or 7 d after regrouping.

## DISCUSSION

### Growth performance, blood cortisol and metabolites

We found that regrouping reduced the growth rate. No comparable studies on the effects of regrouping on the growth performance of dairy young heifers were found in the literature. The regrouped heifers had higher plasma cortisol concentrations 1 d after regrouping, and exhibited more displacement behavior compared to the non-regrouped heifers, suggesting that regrouping caused the stress. Thus, we assumed that the regrouping stress was responsible for the reduced growth observed in this study. In this study, oral Cr and MEL administration recovered the reduced growth observed in the regrouped heifers. The RC and RM heifers had lower circulating cortisol concentrations 1 d after regrouping, and fewer displacement behaviors on d 2 and 3, than RL heifers, indicating that Cr and MEL alleviated the regrouping stress. This alleviation of stress may have contributed to the improved ADG seen in RC and RM heifers. Our study is the first to show that administering oral Cr and MEL improves ADG in regrouped dairy heifers. The Cr supplementation increased the ADG of calves after transportation [14] and under heat stress conditions [22]. The use of absorbed dietary Cr has been suggested to enhance insulin sensitivity [23]. Further study is needed to understand whether increased insulin sensitivity has contributed to the improved ADG associated with Cr supplementation in the regrouped heifers. Administering MEL improved the ADG in transported Jersey calves [21]. The same authors reported that administering MEL may alleviate the negative effects of transport stress on ADG by altering protein metabolism, including skeletal muscle wasting. Oral MEL administration also improved the ADG in transported feeder steers [24]. Notably, our ADG results were obtained during 2 weeks. Whether the improved growth is maintained over a longer time is of interest. No additive effect of the combined Cr and MEL treatment on ADG was observed, indicating that the combined treatment was not beneficial. The TMR was offered as a pen base and all groups consumed all of the feed provided; thus, daily intake was the same among the groups.

We found that the regrouped heifers had a higher cortisol concentration than the non-regrouped heifers 1 d after regrouping. Similarly, cortisol concentrations reportedly increased after mixing unfamiliar calves [1,25].

In this study, administering MEL, Cr, or its combination lowered cortisol concentrations in regrouped heifers 1 d after regrouping. The lowered cortisol concentration in the RM animals 1 d after regrouping suggests that MEL potentially alleviated the stress response in regrouped animals. A decrease in blood cortisol concentration after administering MEL has been observed in other stressed animals, such as transported calves [26] and dehorned calves [27]. Our results were attributed to decreased activation of the hypothalamic-pituitary-adrenal axis after administering MEL [21].

In this study, the Cr treatment decreased cortisol concentrations in the regrouped heifers 1 d after regrouping. The Cr supplementation decreased circulating cortisol concentrations in transported calves [14] and calves under weaning stress [23]. As described above, Cr has been suggested to enhance insulin sensitivity [28]. Further study is needed to determine whether administering Cr affects insulin sensitivity in regrouped heifers.

We found that the regrouped heifers had higher serum NEFA concentrations than the non-regrouped heifers. The increased NEFA concentrations in the regrouped heifers may be explained by increased lipolysis through activation of the hypothalamic-pituitary-adrenal axis [29]. In this study, MEL and Cr administrations decreased the elevated NEFA concentrations in the regrouped heifers. Several nonsteroidal anti-inflammatory drugs have inhibited the degradation of adipose tissue by controlling epinephrine-induced lipolysis [30], which may support the reduced NEFA concentrations observed after administering MEL in this study. Dietary Cr increased the influx of glucose into adipocytes, resulting in reduced release of net fatty acids from adipose tissues in Holstein dairy cattle [30]; this may support the reduced NEFA concentrations in response to Cr treatment seen in this study. Taken together, our results demonstrate that administering MEL and Cr alleviated the increased cortisol and NEFA concentrations in the regrouped heifers, which may reflect reduced regrouping stress. No difference in the NEFA concentration was observed between the RL and RCM treatments, but the reason for the inconsistent result remains unknown.

### Behavioral observations

We found no differences in the duration and frequency of eating and lying among the treatment groups. A previous study reported no difference in eating time after regrouping stress [7].

We found that regrouping caused a marked increase in displacement number. These results confirmed that regrouping stress stimulated displacement behavior in dairy cows [7,31]. We found for the first time that MEL and Cr administrations alleviated the displacement behavior in regrouped heifers at 2 and 3 d after regrouping. The reduced stress with administration of MEL and Cr in the regrouped heifers, which was evidenced by the decreased circulating cortisol concentrations, may be attributed to the decreased displacement behavior.

Displacement behaviors in the regrouped heifers were returned to a similar state as non-regrouped heifers at 7 d after regrouping. Cortisol concentrations were also returned to similar levels as non-regrouped heifers at 7 d after regrouping. It seems that heifers may recover the temporal regrouping stress 7 d after regrouping.

Further studies are needed to examine more blood indices, including immune function and oxidative stress parameters, to understand whether regrouping affects immune function and oxidative stress and whether chromium and MEL administration can restore impaired immune function and alleviate oxidative stress.

## CONCLUSION

Regrouping caused temporal stress, reduced the growth rate, and increased displacement behavior in heifers. Administering Cr and MEL recovered the retarded growth performance and reduced displacement behavior in the regrouped heifers. We conclude that administering both Cr and MEL was effective at alleviating temporal stress in regrouped heifers.

## Figures and Tables

**Table 1:** Effect of regrouping and administration of chromium and meloxicam on the growth performance of Holstein heifers

Items	Treatment^1)^					SEM	p-value

	NL	RL	RC	RM	RCM		
Initial body weight (kg)	197	201	198	197	198	0.01	0.99
Final body weight (kg)	210	209	211	213	217	0.51	0.99
Feed intake^2)^ (kg/d)	8.00	8.00	8.00	8.00	8.00	0.00	-
Average daily gain (kg)	0.94^ab^	0.71^c^	0.90^ab^	1.38^a^	1.42^a^	0.07	<0.01

n = 10/group.

SEM, standard error of the mean.

1) NL, no regrouping and administration of lactose monohydrate; RL, regrouping and administration of lactose monohydrate; RC, regrouping and administration of chromium; RM, regrouping and administration of meloxicam; RCM, regrouping and administration of both chromium and meloxicam.

2) Intake was measured pen-base (10 heifers/pen); intake/heifer was calculated by dividing pen intake by 10 heifers.

a–c Means with different superscripts within a row differ at p<0.05 (Tukey-Kramer test).

**Table 2:** Effect of regrouping and administration of chromium and meloxicam on blood parameters of Holstein heifers

Items	Treatment^1)^					SEM	p-value		
	
	NL	RL	RC	RM	RCM		Treatment	Time	Treatment×time
Cortisol (nmol/L)	0.37	0.48	0.36	0.30	0.34	0.01	0.12	<0.01	<0.01
Glucose (mg/dL)	66.2^ab^	66.1^ab^	64.0^bc^	60.0^c^	70.1^a^	0.51	<0.01	<0.01	<0.01
NEFA (μmol/L)	94.9^b^	114^a^	92.3^b^	87.6^b^	99.8^ab^	2.44	<0.01	<0.01	<0.01

n = 10/group.

SEM, standard error of the mean; NEFA, non-esterified fatty acids.

1) NL, no regrouping and administration of lactose monohydrate; RL, regrouping and administration of lactose monohydrate; RC, regrouping and administration of chromium; RM, regrouping and administration of meloxicam; RCM, regrouping and administration of both chromium and meloxicam.

a–c Means with different superscripts within a row differ at p<0.05 (Tukey-Kramer test).

**Table 3:** Effect of regrouping and administration of chromium and meloxicam on behaviors of Holstein heifers

Items	Treatment^1)^					SEM	p-value		
	
	NL	RL	RC	RM	RCM		Treatment	Time	Treatment×time
Eating duration (min/d)	213	195	183	190	201	4.25	0.35	<0.01	0.14
Lying duration (min/d)	648	677	705	692	696	11.06	0.14	<0.01	0.16
Eating frequency (no./d)	7.61	8.57	8.54	8.30	7.95	0.16	0.23	<0.01	0.06
Lying frequency (no./d)	16.9	14.6	15.27	16.5	15.8	0.31	0.12	<0.01	0.35
Displacement (no./d)	2.64^b^	9.42^a^	5.42^ab^	4.91^ab^	4.57^b^	1.20	0.03	<0.01	0.03

n = 10/group.

SEM, standard error of the mean.

1) NL, no regrouping and administration of lactose monohydrate; RL, regrouping and administration of lactose monohydrate; RC, regrouping and administration of chromium; RM, regrouping and administration of meloxicam; RCM, regrouping and administration of both chromium and meloxicam.

a,b Means with different superscripts within a row differ at p<0.05 (Tukey-Kramer test).

## SUPPLEMENTARY MATERIAL

**Supplementary Table S1**. Ingredient and chemical composition of total mixed ration

**Supplementary Table S2**. Ingredient and chemical composition of concentrate

**Supplementary Table S3**. Ethogram used in this study
