# Supplementary material for: Administration of chromium picolinate and meloxicam alleviates regrouping stress in dairy heifers
Source: Anim Biosci. 2024 Apr 26;37(8):1495–502. doi: 10.5713/ab.24.0104 (PMC11222853; doi:10.5713/ab.24.0104)
Supplement: Supplementary file 1 [file ab-24-0104-Supplementary-Table-1.pdf]

Supplemental table S1. Ingredient and chemical composition of total mixed ration

Item	Percentage
Ingredient, % of DM	
Corn	1.00
Corn gluten feed	9.00
Alfalfa hay	4.00
Oats	1.04
Ryegrass silage	32.0
Probiotics	0.15
Vitamin premix[1]	0.15
Tall fescue, hay	6.00
Beet pulp	5.00
Brewers grain	7.00
Rice straw	8.11
Concentrate[2]	26.6
Total	100
Chemical composition, % of DM, unless otherwise indicated	
DM	64.1
Crude protein	15.9
Ether extract (EE)	3.21
Crude fiber	17.8
Crude ash	8.70
Neutral detergent fiber	47.5
Acid detergent fiber	27.3
Total digestible nutrient	69.4
Digestible energy (DE)[3], Mcal/kg	3.06
Metabolizable energy[4], Mcal/kg	2.39

[1] Vitamin premix contained 5,000,000 IU vitamin A, 1,500,000 IU vitamin D_3, 30,000 IU vitamin E, 2,250 mg Cu, 37,500 mg Fe, 21,000 mg Mn, 75 mg Co, 35,000 mg Zn, 450 mg KIO_3, 70 mg Na_2SeO_3, 15,000 mg Zn-methionine, 30 mg Se yeast, 9,000 mg chelated Zn, 525 mg chelated Cu, 10,000 mg Niacin, 10,000 mg pantothenic acid and 100 mg biotin per kg of additive (provided by Nonghyupsaryo, Busan, Korea).

[2] Detail composition of concentrate is provided in Supplemental Table S2.

[3] Digestible energy (DE) = 0.04409 × total digestible nutrient (%) (NRC. 2001, Nutrient Requirements of Dairy Cattle. 7th ed. Natl. Acad. Sci.).

[4] Metabolizable energy = [1.01 ×(DE) – 0.45] + 0.0046 × (EE – 3) (NRC, 2001).
