# Supplementary material for: Administration of chromium picolinate and meloxicam alleviates regrouping stress in dairy heifers
Source: Anim Biosci. 2024 Apr 26;37(8):1495–502. doi: 10.5713/ab.24.0104 (PMC11222853; doi:10.5713/ab.24.0104)
Supplement: Supplementary file 2 [file ab-24-0104-Supplementary-Table-2.pdf]

Supplemental Table S2. Ingredient and chemical composition of concentrate

Item	Percentage
Ingredient, % of DM	
Corn	19.1
Dried distiller's grains with solubles	7.00
Palm kernel cake	10.0
Corn gluten feed	17.0
Tapioca	5.00
Soybean meal	13.0
Molasses	5.00
Calcium phosphate	0.23
Protease	0.10
Calcium sulfate	0.20
Wheat bran	0.90
Coconut meal	16.0
Corn germ meal	4.00
Limestone	2.00
Salts	0.40
Total	100
Chemical composition, % of DM, unless otherwise indicated	
DM	89.5
Crude protein	20.7
Ether extract (EE)	3.87
Crude fiber	9.27
Crude ash	8.27
Neutral detergent fiber	29.3
Acid detergent fiber	14.0
Total digestible nutrient	80.4
Digestible energy (DE)[1], Mcal/kg	3.54
Metabolizable energy[2], Mcal/kg	3.09

[1] Digestible energy (DE) = 0.04409 × total digestible nutrient (%) (NRC, 2001. Nutrient Requirements of Dairy Cattle. 7th ed. Natl. Acad. Sci.).

[2] Metabolizable energy = [1.01 ×(DE) – 0.45] + 0.0046 × (EE – 3) (NRC, 2001).
